# Spatial‐temporal characteristics of AIDS incidences in Mainland China

**DOI:** 10.1002/iid3.313

**Published:** 2020-06-16

**Authors:** Zhigang Wu, Weining Liang, Weikang Chen, Yanxiang Chang, Yanlong Liu, Xiaodong Liu, Hong Huang, Xuejun Shang

**Affiliations:** ^1^ Department of Andrology, Jinling Hospital, The First School of Clinical Medicine Southern Medical University Nanjing China; ^2^ Department of Andrology The First Affiliated Hospital of Wenzhou Medical University Wenzhou China; ^3^ Center for Health Assessment Wenzhou Medical University Wenzhou China; ^4^ Zhejiang Provincial Key Laboratory of Watershed Science and Health, School of Public Health and Management Wenzhou Medical University Wenzhou China

**Keywords:** empirical orthogonal function, spatial mode, spatial‐temporal decomposition, temporal coefficient

## Abstract

**Object:**

Revealed the spatial‐temporal patterns of acquired immune deficiency syndrome (AIDS) incidences in Mainland China.

**Methods:**

Empirical orthogonal function (EOF) technique was applied to analyze the major spatial distribution modes and the temporal changes of AIDS incidences in Mainland China during 2002‐2017.

**Results:**

The annual average AIDS incidences increased from 0.06 per 100 000 in 2002 to 4.15 per 100 000 in 2017, with an annual average increase of 0.31 per 100 000. The southwest regions were high‐incidence areas, as well as Xinjiang province in the northwest. There were two typical spatial modes. EOF 1 represented an isodirectional spatial pattern that the incidences were relatively high in general, and the fluctuation ranges were relatively high in the southwest and northeast. EOF 2 represented a reverse spatial pattern that the incidences were relatively high (or low) in Guangxi, Yunnan, Xinjiang, Shanghai, and Henan, yet were relatively low (or high) in the remaining regions.

**Conclusion:**

The AIDS incidences in Mainland China were relatively low during 2002‐2010, yet were kept in a relatively high level since 2012. The prevention and control of AIDS need further development, especially in the southwest regions.

## Graphical abstract

Applied the empirical orthogonal function technique to analyze the spatial‐temporal patterns of acquired immune deficiency syndrome incidences in Mainland China, provided decision reference for disease prevention and control.

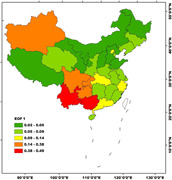

## 1 INTRODUCTION

Many infectious diseases (eg, bilharziasis, malaria) have been controlled effectively along with the development of economy and society and improvement of medication, yet the incidences of some infectious diseases (eg, acquired immune deficiency syndrome [AIDS], gonorrhea) are still keeping up due to various causes such as environmental degradation, unhealthy lifestyles.^1^ Worldwide trends, such as demographic aging, urbanization, and the climate change, have contributed to the emerging communicable diseases (eg, human avian influenza, Ebola virus disease, COVID‐19), as well as the variety of the emergence, resurgence, and spread of infectious diseases.^2^ Infectious diseases are still menacing to people's health, how to effectively prevent and control remains challenges.^3^, ^4^

Most infectious diseases have both spatial and temporal and attributes,^5^, ^6^ and the occurrence and spread have characteristics of diversity, complexity, and spatiotemporal heterogeneity.^7^, ^8^ Identifying the spatial‐temporal of the infectious diseases in one of the emphases and difficulties in the field of spatial epidemiology. Over the years, the development of geographic information systems and spatial analysis techniques provide very important supports for spatial epidemiology. The widely used methods for analyzing the spatial‐temporal patterns of infectious diseases include space‐time scan statistics, space‐time clustering, Spatiotemporal autocorrelation analysis,^9^, ^10^, ^11^ and so on. These methods/techniques are playing key roles in identifying the characteristics of incidences and epidemic trends of infectious diseases, providing an important basis for decision‐making on prevention and control, as well as public health emergency.^12^

Empirical orthogonal function (EOF), also known as eigenvector analysis, is a technique for analyzing the structural features and extracting the feature quality of matrix data. EOF is one of the important and universal techniques for spatial‐temporal analysis in meteorology and climatology,^13^ and has been also widely applied in the field of geology,^14^ oceanology,^15^ and environmental sciences,^16^ and so on. AIDS is one of the most destructive diseases in human history and is still threatening public health in many countries and regions.^17^, ^18^ The aim of this paper is to apply the EOF technique in analyzing the spatial‐temporal patterns of AIDS incidences in Mainland China during 2002‐2017, to provide decision reference for disease prevention and control, and to enrich the spatial‐temporal analysis methods for spatial epidemiology.

## 2 MATERIALS AND METHODS

### 2.1 Data source

**Figure 1:**
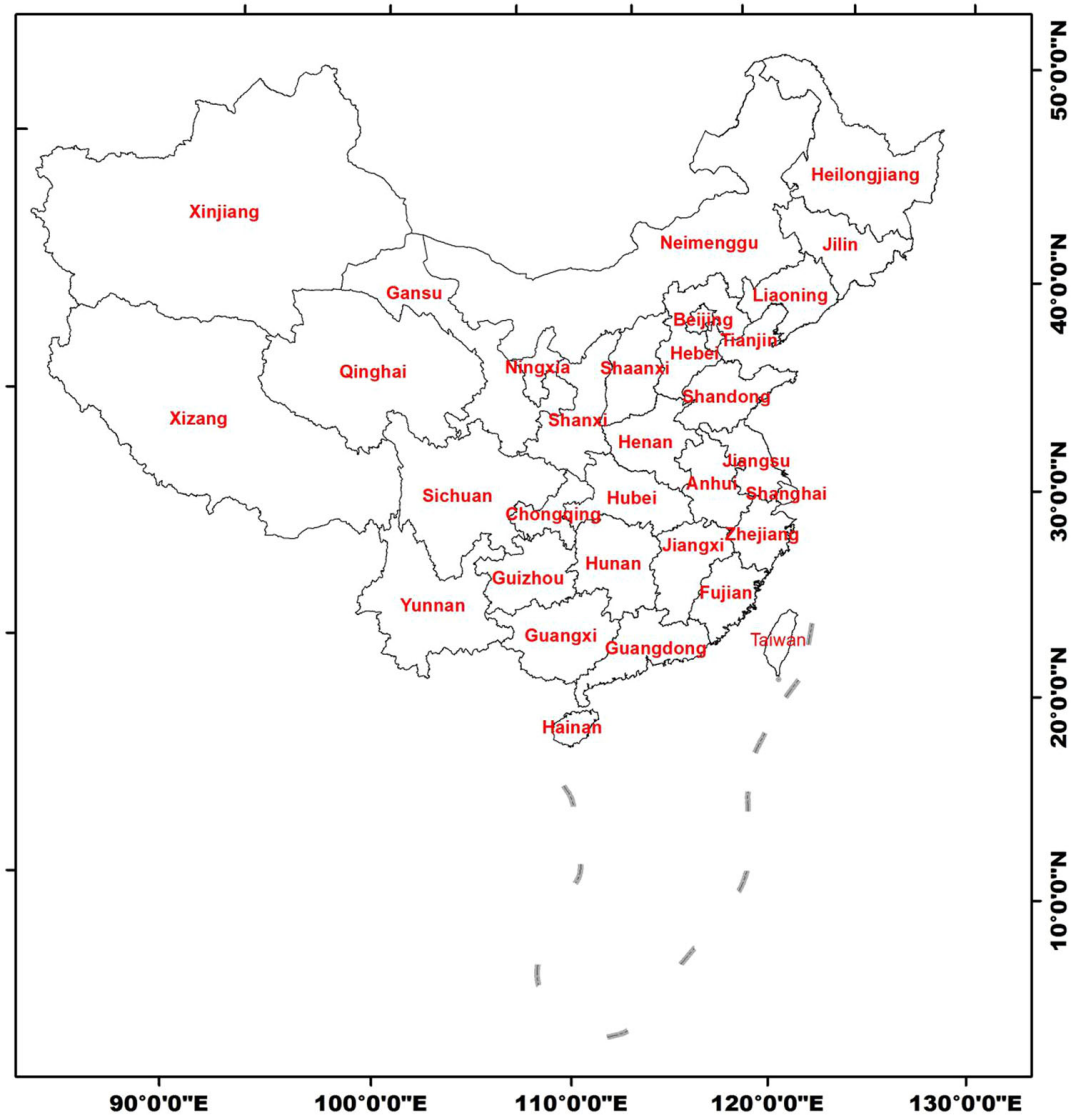
The geographical location of study area

Datasets of the paper were obtained from the China Statistical Yearbooks Database (http://tongji.cnki.net/kns55/index.aspx). The study area covering 31 provincial administrative regions in Mainland China (Hong Kong, Macao, and Taiwan data not shown) (Figure 1). The major materials were the annual AIDS incidences in the 31 provincial administrative regions during 2002‐2017.

### 2.2 A brief introduction on EOF

EOF decomposes the spatial‐temporal data matrix into two parts of the eigenvector and principal component, which are also called spatial mode and temporal coefficient, respectively. The eigenvector reflects the major spatial distribution characteristics, while the absolute value of the eigenvector indicates the spatial variations. For a spatial‐temporal matrix *X*_*m* × *n*_ (*m* is the sample size, *n* is the time length), the major calculation procedure of EOF are as follows^19^:

(1) Calculating the crossed product of matrix *X*_*m* × *n*_ and its transposed matrix
  (1) $$C_{m\times m}=\frac{1}{n}X\times X^{T},$$
  where *X*_*m* × *m*_ has been departured and *C*_*m* × *m*_ is the covariance matrix.
(2) Calculating the eigenvalue (*λ*_1_, …, *λ_m_*) and the eigenvector *V*_*m* × *m*_, where
  (2) $$C_{m\times m}\times V_{m\times m}=V_{m\times m}\times E_{m\times m}.$$
*E*_*m* × *n*_ is a diagonal matrix
  (3) $$E=\left[\begin{array}{cccc} \lambda_{1} & 0 & \cdots & 0\\ 0 & \lambda_{2} & \cdots & 0\\ \vdots & \vdots & \vdots & \vdots \\ 0 & 0 & \cdots & \lambda_{m} \end{array}\right].$$
(3) Calculating the principal component
  (4) $$PC_{m\times n}=V_{m\times m}^{T}\times X_{m\times n},$$
  where each row in *P*_*m* × *n*_ represents the temporal coefficient of the corresponding eigenvector.
(4) Calculating the variance contribution
  (5) $${SM}_{k}=\frac{\lambda_{k}}{\sum_{i=1}^{m}\lambda_{i}}\times 100\%,$$
  where, SM_*k*_ represents the variance contribution the *k*th eigenvector.
(5) Significance test
  The error range of an eigenvalue at a 95% level is
  (6) $$\Delta \lambda =\lambda \sqrt{\frac{2}{N^{*}}},$$
  where N* is the degree of freedom. The eigenvalues are sort from largest to smallest, and if the error ranges of two neighboring eigenvalues are not overlapping, it could be considered as passing the significant test.

## 3 RESULTS AND DISCUSSION

### 3.1 Basic information

**Figure 2:**
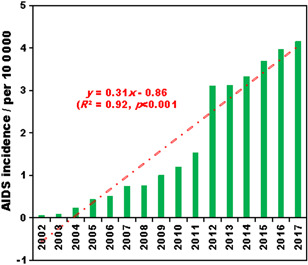
Annual average AIDS incidences in Mainland China during 2002‐2017. AIDS, acquired immune deficiency syndrome

**Figure 3:**
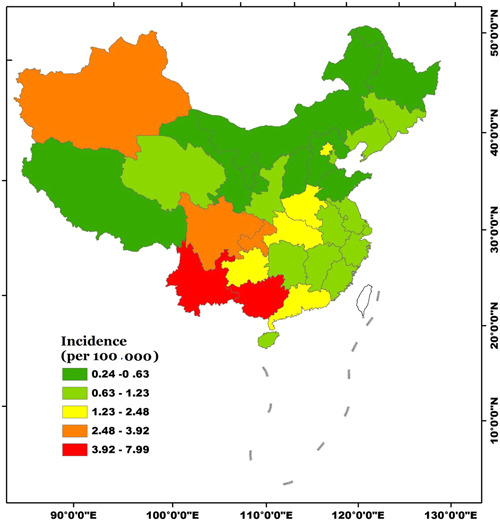
The annual average of AIDS incidences of the provinces in Mainland China during 2002‐2017. AIDS, acquired immune deficiency syndrome

During 2002‐2017, the annual average AIDS incidences in Mainland China increased from 0.06 per 100 000 in 2002 to 4.15 per 100 000 in 2017, with an annual average increase of 0.31 per 100 000 (Figure 2). It should be noticed that the incidences jumped from 1.53 per 100 000 in 2011 to 3.11 per 100 000 in 2012, and kept at a relatively high level since 2012 (Figure 2). The annual average AIDS incidences in the 31 provincial administrative regions during 2002‐2017 were calculated, and the mean, minimum, and maximum were 1.62, 0.25, and 7.99 per 100 000, respectively. By means of the natural breakpoint method, the annual average AIDS incidences in the 31 regions during 2002‐2017 were divided into five classes (Figure 3). Class I included Guangxi and Yunnan, while class II included Xinjiang, Sichang, and Chongqing. These five regions could be considered as the high‐incidence (Figure 3). Class III had Guizhou, Henan, Guangdong, Hubei, and Beijing, and these five regions could be considered as moderate‐incidence (Figure 3). The remaining 21 regions were belonging to class IV and V with relatively low incidences (Figure 3). For spatial distribution, the southwest region could be considered as a high‐incidence area, as well as the northeast region. These results were consistent with previous study.^18^

### 3.2 Spatial patterns of AIDS incidence rate

There are a number of spatial modes decomposed by EOF, yet only the several important modes are used in practice.^19^ The spatial‐temporal data matrix of AIDS incidences of Mainland China during 2002‐2017 was decomposed by EOF, and the variance contributions of the first five eigenvectors were showed. The first eigenvector (EOF 1) and second eigenvector (EOF 2) contributes 92.2% and 6.6%, respectively, and the cumulative variance contribution was 99.3% (Table 1). The error ranges of the eigenvalue of EOF 1 and EOF 2 were 91.8 to 154.4 and 6.6 and 11.0, respectively (Table 1). It could be concluded that the first two spatial modes could represent the spatial patterns of AIDS incidences in Mainland China.

**Table 1:** The variance contributions and cumulative variance contributions of the first five eigenvectors from EOF analyzing on AIDS incidences in Mainland China during 2002‐2017

Spatial mode	Eigenvalue	Variance contribution/%	Cumulative variance contribution/%	Eigenvalue error	
				Lower limit	Upper limit
EOF 1	123.1	92.2	92.2	91.8	154.4
EOF 2	8.8	6.6	98.8	6.6	11.0
EOF 3	0.7	0.5	99.3	0.5	0.8
EFO 4	0.4	0.3	99.6	0.3	0.5
EOF 5	0.3	0.2	99.8	0.2	0.4

Abbreviation: EOF, empirical orthogonal function.

**Figure 4:**
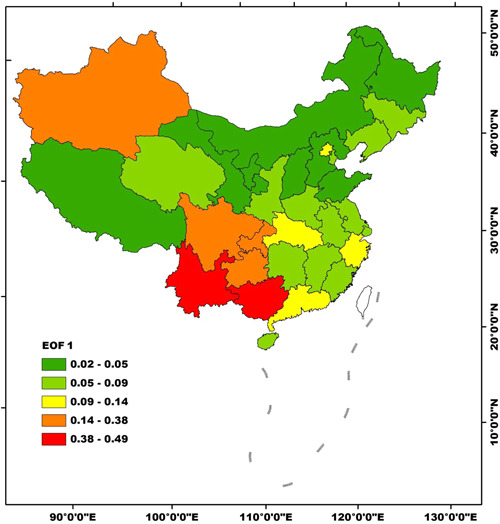
Spatial distributions of EOF 1 of AIDS incidences in Mainland China during 2002‐2017. AIDS, acquired immune deficiency syndrome; EOF, empirical orthogonal function

The variance contribution of EOF 1 was 92.2% and much higher than the others, indicating it was the major typical spatial mode of AIDS incidences in Mainland China. Meanwhile, the eigenvector of EOF 1 was positive in all of the 31 regions, and high‐value regions were Guangxi, Yunan, Sichuan, Chongqing, and Guizhou in the southwest China, as well as in Xinjiang in the northeast China (Figure 4). Hence, EOF 1 represents an isodirectional spatial pattern. In the case of EOF 1 was in charge, the AIDS incidences in all of the 31 regions were relatively high or low consistently, yet the fluctuation ranges were relatively high in the southwest and northeast China.

**Figure 5:**
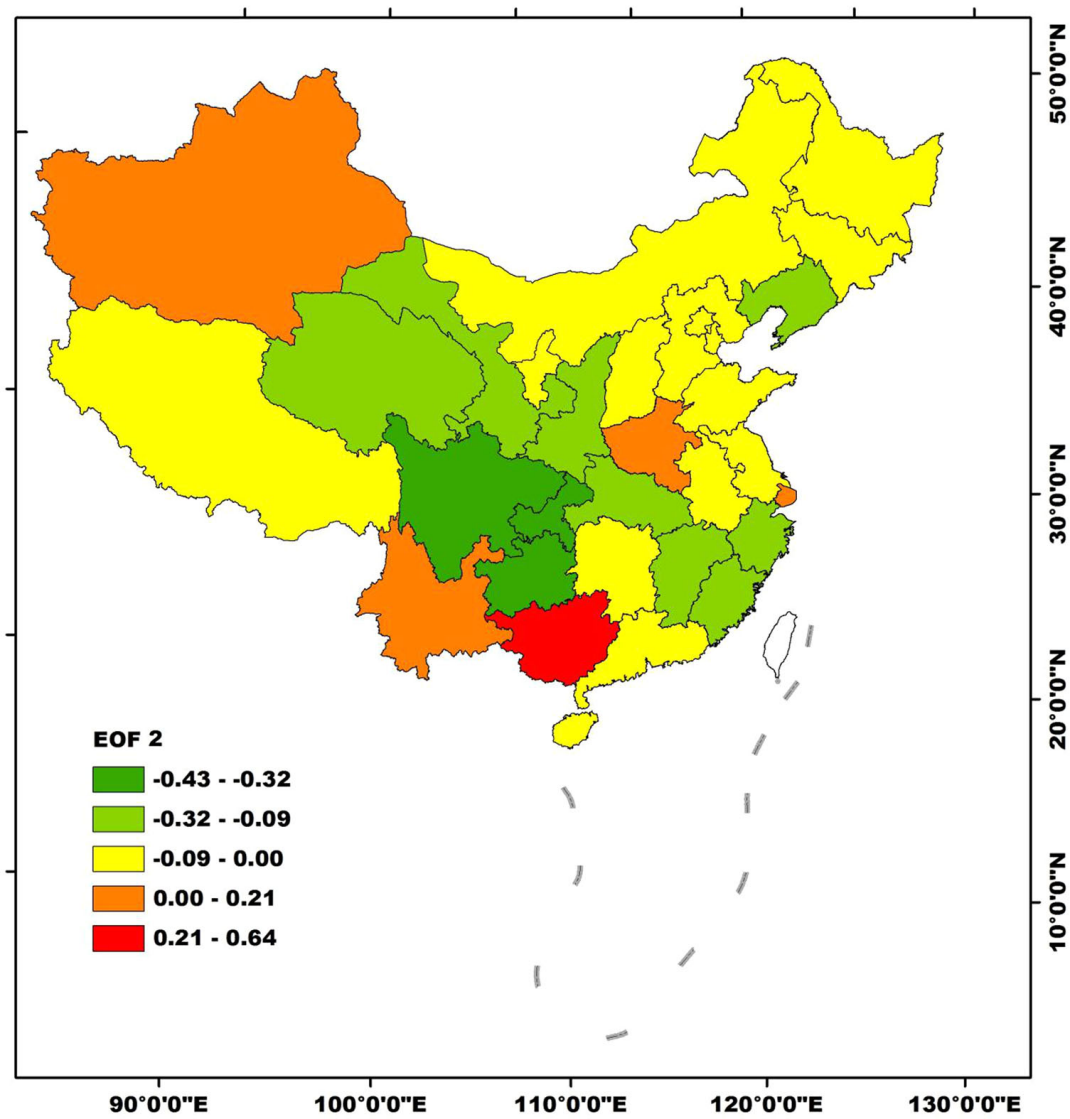
Spatial distribution of EOF 2 of AIDS incidences in Mainland China during 2002‐2017. AIDS, acquired immune deficiency syndrome; EOF, empirical orthogonal function

The variance contribution of EOF 2 was 6.6% and was also one of the typical spatial modes. The eigenvector of EOF 2 was positive in Guangxi, Yunnan, Xinjiang, Shanghai, and Henan, yet it was negative in the other regions (Figure 5). The high‐value center of positive values was in Guangxi, while the low‐value centers of negative values were Sichuan, Chongqing, and Guizhou (Figure 5). EOF 2 also represents a typical spatial mode that the AIDS incidences were reverse distribution. In the case of EOF 2 was responsible, AIDS incidences were relatively high (low) in Guangxi, Yunnan, Xinjiang, Shanghai, and Henan yet were relatively low (high) in the remaining regions (Figure 5).

In general, there were two typical spatial modes of AIDS incidents in Mainland China, yet which mode was in charge in each year should be judged by the temporal coefficients which were discussed in the next subsection.

### 3.3 Temporal patterns of AIDS incidence rate

**Figure 6:**
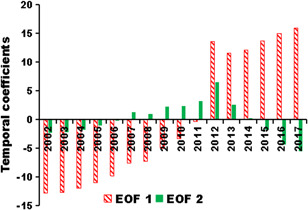
Temporal coefficients of EOF 1 and EOF 2 of AIDS incidence rate in Mainland China during 2002‐2017. AIDS, acquired immune deficiency syndrome; EOF, empirical orthogonal function

Each spatial has a corresponding temporal coefficient series that reflects the temporal changes of the spatial distribution characteristics of AIDS incidences. Although there might be several typical spatial modes during the long‐term period, yet only one typical spatial mode can be appearing in 1 year. The magnitude of the absolute value of the principal component indicates the typical degree of the corresponding spatial mode.^20^ In consideration that EOF 1 and EOF 2 were the most typical spatial modes, the temporal coefficients were calculated and showed in Figure 6. The absolute value of the principal component of EOF 2 was higher than EOF 1 in 2011, yet were lower in the other study years. Therefore, EOF 2 was the typical spatial model in 2011, while EOF 1 was in charge of the remaining years (Table 2).

**Table 2:** The temporal changes of the spatial modes of AIDS incidences in Mainland China during 2002‐2017

Typical spatial mode	Time period	Spatial distribution patterns
EOF 1	2002‐2010	Relative low in Mainland China, but was increasing
EOF 2	2011	Relative high in Guangxi, Yunnan, Xinjiang, Shanghai, and Henan, relative low in the other regions
EOF 1	2012‐2017	Relative high in Mainland China

The temporal coefficients of EOF 1 were negative in 2002‐2011, and increasing from −12.9 in 2002 to −0.4 in 2011 and then were jumping to a high and positive level since 2012 (Figure 3C). Meanwhile, the temporal coefficients of EOF 2 were negative in 2002‐2014, positive in 2007‐2014, and negative in 2015‐2017 (Figure 6). If the values of the spatial mode are positive (negative) in a certain region, and the temporal coefficient in a certain year is positive (negative), the AIDS incidences in this region this year are relatively high.^19^ By contrast, if the values of the spatial mode are positive (negative) in a certain region, and the temporal coefficient in a certain year is negative (positive), the AIDS incidence rate in this region this year is relatively low. The temporal changes of the spatial modes of AIDS incidences were summarized and listed in Table 2. In 2002‐2010, EOF 1 was the typical spatial mode, the temporal coefficients were negative and were increasing, indicating that AIDS incidences in Mainland China were relatively high yet increasing during this time period. In 2011, EOF 2 was the typical spatial mode, the temporal coefficient was positive, meaning that AIDS incidences in Guangxi, Yunnan, Xinjiang, Shanghai, and Henan were relatively high, yet were relatively low in the other regions. In 2012‐2017, EOF 1 was the typical spatial mode, and AIDS incidences were keeping at a relatively high level.

## 4 CONCLUSIONS

The annual average AIDS incidences in Mainland China were increasing during 2002‐2017, and keeping at a high level since 2002. The southwest regions were high‐high clustering areas for AIDS incidence rate, and Xinjiang in the northeast is also one high‐incidence area. Even though the Government and other forces have done a lot of work, the situation should not be too optimistic, the prevention and control of AIDS need further development.

## CONFLICT OF INTERESTS

The authors declare that there are no conflict of interests.

## Data Availability

The data used in this article are available from the corresponding author upon request.
